# Stimuli-responsive nanocarriers for therapeutic applications in cancer

**DOI:** 10.20892/j.issn.2095-3941.2020.0496

**Published:** 2021-06-15

**Authors:** Xubo Zhao, Jie Bai, Wenjing Yang

**Affiliations:** 1 Green Catalysis Center, and College of Chemistry, Zhengzhou University, Zhengzhou 450001, China; 2 Department of Anesthesiology, The First Affiliated Hospital of Zhengzhou University, Zhengzhou 450052, China

**Keywords:** Nanocarriers, therapeutic agent, nanomedicine, stimuli-responsiveness, cancer therapy

## Abstract

Cancer has become a very serious challenge with aging of the human population. Advances in nanotechnology have provided new perspectives in the treatment of cancer. Through the combination of nanotechnology and therapeutics, nanomedicine has been successfully used to treat cancer in recent years. In terms of nanomedicine, nanocarriers play a key role in delivering therapeutic agents, reducing severe side effects, simplifying the administration scheme, and improving therapeutic efficacies. Modulations of the structure and function of nanocarriers for improved therapeutic efficacy in cancer have attracted increasing attention in recent years. Stimuli-responsive nanocarriers penetrate deeply into tissues and respond to external or internal stimuli by releasing the therapeutic agent for cancer therapy. Notably, stimuli-responsive nanocarriers reduce the severe side effects of therapeutic agents, when compared with systemic chemotherapy, and achieve controlled drug release at tumor sites. Therefore, the development of stimuli-responsive nanocarriers plays a crucial role in drug delivery for cancer therapy. This article focuses on the development of nanomaterials with stimuli-responsive properties for use as nanocarriers, in the last few decades. These nanocarriers are more effective at delivering the therapeutic agent under the control of external or internal stimuli. Furthermore, nanocarriers with theranostic features have been designed and fabricated to confirm their great potential in achieving effective treatment of cancer, which will provide us with better choices for cancer therapy.

## Introduction

**Figure 1:**
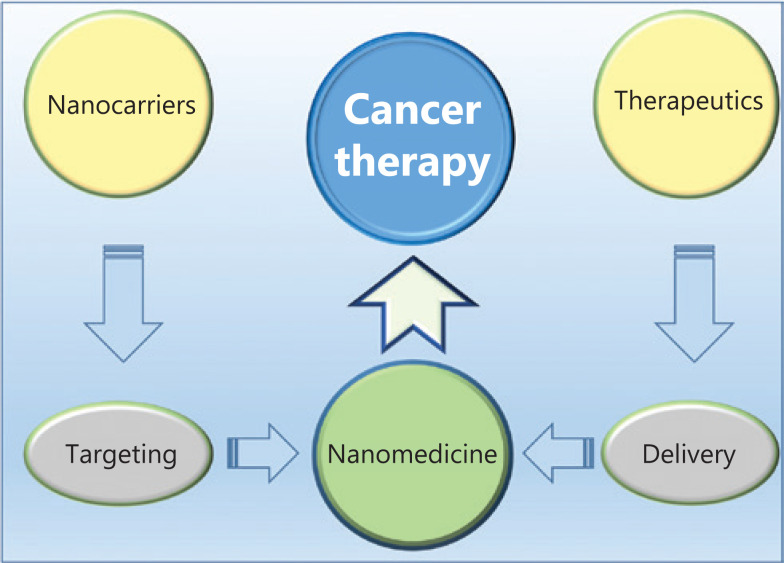
Schematic illustration of the formation of nanomedicine for cancer therapy.

Cancer has always been a deadly disease. By the end of the century, cancer will be the disease with the highest mortality rate worldwide and the greatest obstacle to overcome in aging humans. In 2018, cancer was responsible for 9.6 million deaths worldwide^1^. Among the cancer treatments, chemotherapy has become increasingly important because of its effectiveness. Although chemotherapy might be an effective treatment for cancer, its efficacy is hindered by poor targeting, drug tolerance, a low therapeutic index, and adverse drug reactions^2^. Recently, the development of nanotechnology has provided a once-in-a-lifetime opportunity to improve the efficacy of chemotherapy. Innovative nanocarriers improve the function and efficacy of chemotherapy, resulting in the widespread application of nanotechnology. Through a combination of nanocarriers and therapeutics, nanomedicine (the medical application of nanotechnology) has been successfully used to treat cancer in recent years (**Figure 1**)^3,4^. Nanocarriers have been proposed to serve as a new drug delivery strategy in recent years, because they achieve the selective accumulation of drugs in the tumor tissue, reduce the side effects of therapeutic agent, and improve the efficacy of chemotherapy through active and passive targeting strategies^5–7^. Typically, nanocarriers have been rationally designed based on organic polymers because of their negligible cytotoxicity, biocompatibility, and easy modification. Although polymer-based nanocarriers have the aforementioned advantages for drug delivery, they are limited by the premature leakage of loaded-drugs, irreversible deformation, and inherently inferior stability; therefore, these limitations must be overcome for cancer therapy. Hybrids, another type of effective nanocarrier, comprise inorganic components and organic polymers with excellent stability, high drug-encapsulating capability, and theranostic features, and thus have been widely used to treat cancer in recent years^5,8,9^.

**Figure 2:**
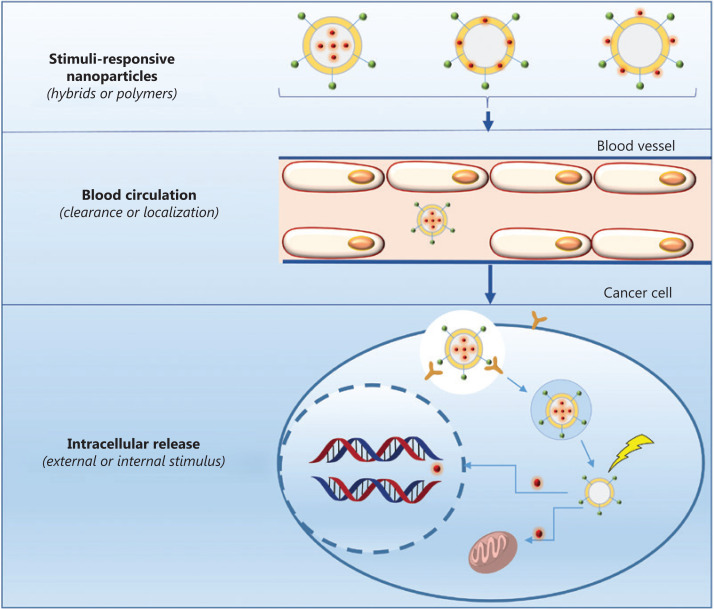
Delivery pathway of therapeutic agents through nanocarriers.

Although the components and structures of nanocarriers vary substantially, the drug transport strategies used for systemic delivery and drug release at the target sites are similar because of similar interactions between the network of nanocarriers and drugs. Upon intravenous administration, the nanomedicine encounters numerous barriers, such as intratumor pressure, aberrant tumor vasculature, a mononuclear phagocyte system, and drug resistance^10^ as shown in **Figure 2**. Once the nanomedicine overcomes these pathophysiological barriers, it is able to achieve its therapeutic potential. Nanomedicine has achieved excellent efficacy in clinical settings, when compared with conventional chemotherapeutics. Of course, this efficacy is based on the stimuli responsiveness of nanocarriers (**Figure 2**). These stimuli are divided into internal and external stimuli, according to the sources of stimuli, as presented in **Figure 2**. Compared with normal tissues, tumor tissues have many unique features, such as elevated glutathione (GSH) levels, overexpressed enzymes, and an acidic pH, which have been used to fabricate nanocarriers (**Figure 6**). In addition to these internal stimuli, some external stimuli such as light, heat, and ultrasound, have also been exploited to design nanocarriers (**Figure 7**). Based on the aforementioned stimuli, nanocarriers for cancer treatment have been rationally designed with different responsive structures, from polymers to hybrids, and have been designed to exhibit various drug-release mechanisms upon exposure to internal and external stimuli. Here, we will discuss the different constituents of nanocarriers, systematically summarize several stimuli that promote drug release from nanocarriers, and analyze the success of nanocarriers as a nanomedicine, as well as their future prospects.

## Nanocarriers

Through the combination of nanotechnology and therapeutics, nanomedicine has been successfully used to treat cancer in recent years. Therapeutic agents, including cisplatin, docetaxel, paclitaxel, 5-fluorouracil, mitoxantrone, cantharidin, irinotecan, and doxorubicin are usually considered active payloads in nanomedicine^4,11–17^, while the nanocarrier serves as a vehicle to deliver these active payloads to target sites for drug delivery. As shown in **Figure 2**, nanocarriers are divided into the following 3 types based on the location of the therapeutic agents within these structures: (1) therapeutic agents are encapsulated into the internal core of nanocarriers; (2) therapeutic agents are loaded on the shell of nanocarriers; and (3) therapeutic agents are anchored on the external surface of nanocarriers. Compared with therapeutic agents, different nanocarriers are important because of their ability to encapsulate therapeutic agents and deliver the drug to the target site, as well as their biocompatibility and biodegradability. Therefore, nanocarriers have been proposed to serve as a new drug delivery strategy in recent years, because they achieve the selective accumulation of drugs in tumor tissue, reduce the side effects of therapeutic agents, and improve the efficacy of chemotherapy through active and passive targeting strategies. Therefore, nanomedicine has achieved great progress compared with conventional chemotherapy drugs in clinical settings.

## Polymer-based nanocarriers

Polymer-based nanocarriers are currently used to meet the fundamental challenge in drug delivery: They provide a sufficient dosage of therapeutic agents, overcoming pathophysiological barriers *in vivo*, and release it at the tumor site without side effects (**Figure 4**). Since the 1960s, polymer-based nanocarriers have been extensively used for the delivery of therapeutic agents^18–21^. We have witnessed an evolution in polymer-based nanocarriers for cancer therapy over the last few decades. The emergence of polymer-based nanocarriers has provided a new perspective on the treatment of cancer. Furthermore, the need to improve the delivery efficiency and to develop safer nanocarriers has led to extensive investigations of synthetic and natural polymers.

Polymer-based nanocarriers, including synthetic and natural polymers, are the most widely investigated nanocarriers^4,22^. Some typical natural polymers used for this purpose are chitosan, alginate, and hyaluronic acid. As an example, Zhao et al.^22^ prepared a graphene oxide-based drug delivery system containing chitosan as an external shell *via* the classic self-assembly method. The resulting nanocarriers avoided the premature release of therapeutic agents in normal extracellular media because of the introduction of the chitosan shell, and the release of therapeutic agents was subsequently accelerated because of the detachment of the chitosan shell in acidic media^22^. Synthetic polymers have been increasingly used for drug delivery because of their biodegradability, designability, universality, and biocompatibility properties. Based on these qualities of synthetic polymers, many of the synthetic polymer-based nanocarriers are commonly used to deliver therapeutic agents to tumors. Sun et al.^23^ reported a polymeric micelle containing a typical monomethoxy polyethylene glycol stealth component to avoid the rapid clearance of nanocarriers due to an immunological response and to improve anticancer drug delivery. Liao et al.^24^ also reported a polymer-based nanocarrier that showed a charge-conversion behavior and synergistic effect *in vitro*.

The structure and function of polymer-based nanocarriers must be regulated to improve the delivery of therapeutic agents. An understanding of the interaction between the structure and function of a polymer will contribute to the design of new and effective polymer-based nanocarriers. Polymer-based nanocarriers have achieved substantial progress from their humble beginnings to state-of-the-art tailored molecules that take advantage of the diversity of polymer topology. Importantly, chemical innovation has been widely used to optimize the structure and function of polymers in the last few decades. Researchers have expended tremendous effort into the development of synthetic methodologies for polymers, including ring opening metathesis polymerization, ring-opening polymerization, reversible addition-fragmentation chain transfer, atom transfer radical polymerization, and living anionic polymerization^7^.

## Hybrid-based nanocarriers

**Figure 3:**
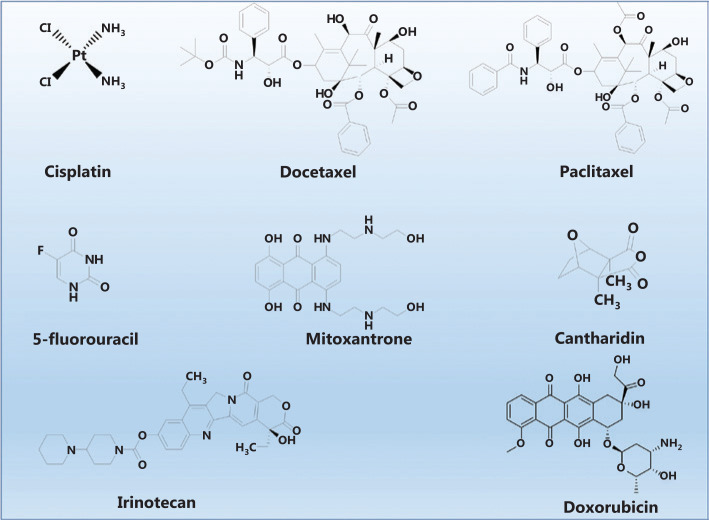
Examples of common therapeutic agents used in drug delivery for cancer therapy.

Although polymer-based nanocarriers possess many advantages for drug delivery, they suffer from poor drug loading, premature leakage of the drug, and inherent inferior stability; therefore, these drawbacks must be addressed to increase the therapeutic effect. Hybrids, another type of effective nanocarrier, comprise inorganic substances and organic polymers with excellent stability and a high drug-encapsulating capability, and thus have been extensively used to deliver many therapeutic agents (**Figure 3**). Poor water solubility limits the bioavailability of these anti-cancer agents and may impede the development of cancer treatments. Zhao and colleagues have performed many systematic studies of the effect of the introduction of inorganic components on enhancing the performance of polymer-based nanocarriers, namely, hybrid-based nanocarriers, to solve this dilemma^3,8,9,25^. As an example, Zhang et al.^26^ also observed an increase in the stability of polymer-based nanocarriers following the introduction of inorganic silica monomer. Miao et al.^3^ reported the innovative preparation of hybrid-based nanocarriers through the formation of manganese oxide, which induced the self-assembly of block copolymers to increase the stability of polymer-based nanocarriers. In this design, the introduction of MnO_2_ increase the structural stability of hybrid-based nanocarriers, and these hybrid-based nanocarriers respond to GSH and weak acidic conditions to unload their cargos. As a result, the hybrid-based nanocarrier is an effective strategy for cancer therapy.

Regardless of the types of inorganic materials used, most hybrid-based nanocarriers are modified by poly (ethylene glycol) (PEG) or other hydrophilic polymers. PEG is one of the most commonly used polymers in drug delivery because of its biocompatibility, hydrophilicity, and shielding property^27^. Thus, an ideal approach is to use PEG to modify the external surface of nanocarriers and avoid the nonspecific uptake by normal cells, leading to enhanced low fouling properties^28,29^. Extensive effort has been devoted to the development of hybrid-based nanocarriers through the introduction of PEG. In a representative example, a multifunctional silicon-based nanostructure was designed and prepared for tumor-targeted multimodal imaging-guided photothermal therapy^30^. After the surface was modified with PEG, the hybrid-based nanocarriers displayed increased biocompatibility^31^. Taking advantage of the introduction of gold nanoparticles, these hybrid-based nanocarriers were suitable for specifically ablating tumors under the guidance of multimodal imaging^30^.

In recent years, hybrid-based nanocarriers have become popular due to the introduction of organic materials, resulting in excellent stability, highly controllable drug release, and multimodal imaging. The structures of hybrid-based nanocarriers should be precisely controlled to achieve more efficient therapy. Moreover, a combination of diagnosis and treatment will likely be promoted, based on the rapid development of hybrid-based nanocarriers, which leads to the increased use of theranostics in the field of tumor treatment. As mentioned above, hybrid-based nanocarriers incorporating theranostic features have been designed and fabricated to confirm their great potential for achieving the accurate treatment of cancer, which will provide us more choices for cancer therapy. Therefore, hybrid-based nanocarriers are an important direction of future development.

## Stimuli-responsive nanocarriers

From the perspective of cancer therapy, an ideal nanomedicine would cure cancer without causing any side effects. Unfortunately, many therapeutic agents kill both cancerous and healthy cells during treatment. As a result, patients usually suffer from severe side effects that further reduce their quality of life. Scientists and medical professionals aim to improve on the precision of therapeutic agents to address these issues. Numerous new drug delivery strategies have been proposed in recent years, such as stimuli-responsive nanocarriers, which achieve the selective release of therapeutic agents in tumor tissues upon exposure to various internal or external stimuli. Compared with normal tissues, tumor tissues have many unique features, such as an elevated GSH level, overexpressed enzymes, and an acidic pH, which have been applied to fabricate nanocarriers. In addition to these internal stimuli, some external stimuli, such as light, heat, and ultrasound, have also been exploited to design nanocarriers.

## The pH-responsive nanocarriers

**Figure 4:**
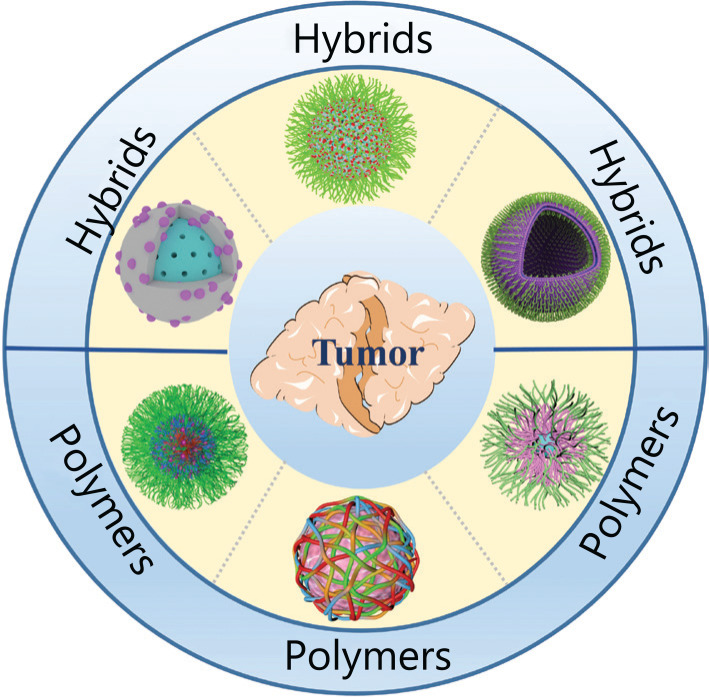
Summary of common nanocarriers used to treat cancer.

Many pH-responsive nanocarriers been widely used in tumor therapy. The major factor in designing pH-responsive nanocarriers is that the extracellular pH in tumor microenvironments ranges from 5.8 to 7.2, and the pH in lysosomes or endosomes is approximately 5.5, both of which are more acidic than the pH (∼7.4) of normal tissues^31,32^. Therefore, both the extracellular environment and intracellular endosomes provide a good internal stimulus for drug release. **Figure 4** illustrates some major pH-responsive nanocarriers that have been used to design nanocarriers for cancer therapy. The pH-responsive mechanism of nanocarriers is attributed to either the degradation of acid-cleavable bonds or protonation of typical groups^31,32^. Based on these mechanisms, in recent years a wide range of pH-responsive nanocarriers have been designed and developed with improved spatiotemporal control of payload delivery with enhanced efficacy^31,32^.

The ubiquitous acidic extracellular microenvironment of the tumor has attracted increasing attention and has been utilized to design and fabricate pH-responsive nanocarriers that selectively deliver the therapeutic agent to tumor sites (**Figure 6**). Dong et al.^33^ prepared a tumor pH-responsive polymer containing tetrazine groups, which were unreactive in micelles in the blood circulation, but were activated in response to the tumor pH and induced micelle disassembly. The ubiquitous acidic extracellular microenvironment presents a general strategy for drug delivery in cancer therapy. The pH-sensitive charge-conversion in the design of nanocarriers is important because it enhances cellular uptake^34^. Liao et al.^24^ reported on prodrug-based systems with charge-conversion for a co-delivery complex used in combination cancer therapy, which exhibited a higher cell-killing performance. The benzoic imine bond is increasingly being used as a pH-responsive building block in treatments for cancer^35^. The pH-responsive nanocarriers also have important applications in the treatment of diseases other than cancer. As an example, Puglisi et al.^36^ prepared pH-responsive nanocarriers as smart cyclodextrin-releasing agents through the introduction of benzoic imine bond. These pH-responsive nanocarriers showed a pH-dependent morphological transformation and represented a promising potential therapeutic tool in the treatment of cholesterol-associated conditions^36^. The acid-labile hydrazone bond has also attracted increasing attention in recent years. A pH-responsive nanocarrier has been developed based on the acid-labile hydrazone bond that maintains its stability, minimizes the payload leakage in blood circulation, and exhibits the pH-sensitive release of drug in cancer cells^37^. Moreover, this pH-responsive nanocarrier was easily traced during drug delivery because of the introduction of a hydrophobic tetraphenylethene-based fluorophore. In a representative example, a novel water-soluble pH stimuli-responsive fluorescent copolymer of poly [polyethylene glycol methyl ether methacrylate-*b*-(methylacryloylhydrazide-*co*-*N*′-Rhodamine 6G-ethyl-acrylamide)] was designed through reversible addition-fragmentation chain transfer (RAFT) polymerization as a pH-responsive nanocarrier^38^. The pH-responsive nanocarriers easily formed as polymeric micelles with diameters of approximately 100 nm in an aqueous solution. Increased efficiency of the delivery of the as-loaded drug was achieved due to the cleavage of the acylhydrazone linkage bond between the nanocarrier and therapeutic agent in an acidic environment^38^. As a result, its structural stability in neutral media and acid-sensitive cleavage in the acidic environment not only avoided the premature leakage of the therapeutic agent from nanocarriers during circulation in the blood, but also increased drug release and improved the therapeutic efficacy against cancer.

Chitosan is a pH-responsive natural polymer with a pKa of approximately 6.5, which has been extensively considered in biomedical applications, particularly in drug delivery^22,34,39,40^. Thus, the slightly acidic condition protonates chitosan and increases its water solubility. For instance, a chitosan-based micelle was designed and fabricated as a smart pH-responsive nanocarrier by modulating the molecular weight of chitosan and feeding ratio of polyethylene glycol^40^. This pH-responsive nanocarrier exhibited a pH-sensitive “off-on” switch due to the solubility of chitosan in media with different pH values. Benefiting from the pH-sensitive “off-on” switch, the pH-responsive nanocarrier exhibited drug leakage-free behavior in a physiological environment, while achieving rapid drug release in the intracellular microenvironment^40^. As another example, pH-sensitive polyelectrolyte-based hollow microspheres with hepatocyte-targeting functions were designed through the layer-by-layer assembly of sodium hyaluronate and chitosan^39^. The pH-sensitive nanocarriers presented pH-sensitive drug release because of the protonation of chitosan under acidic conditions. Furthermore, the pH-responsive nanocarriers showed pH-sensitive orange-yellow fluorescence, which is useful in bioimaging applications in cancer therapy^39^.

Another type of microenvironment that may trigger drug release is the weakly acidic condition inside the endosomes, which has been used to trigger drug release from nanocarriers by the diphtheria toxin endosomolytic module, which functions by generating defects in membranes^41^. Additionally, nanocarriers have been synthesized *in situ* for drug loading to develop nanomedicines^42^.

As mentioned above, the ability of pH-responsive nanocarriers to release their therapeutic agents is mainly determined by the localized pH in tumor sites. With advances in nanotechnology, pH-responsive nanocarriers have already become a useful strategy to accurately distinguish tumor tissues. Notably, pH-responsive nanocarriers should exhibit excellent structural stability in neutral media, followed by the acid-sensitive cleavage of nanocarriers in an acidic environment to release therapeutic agents and improve the therapeutic efficacy for cancer therapy. Therefore, a pH-responsive nanocarrier is an ideal approach for the release of therapeutic agents in the acidic environment of tumor tissues.

## Redox-responsive nanocarriers

The level of GSH is a key marker of tumor tissues compared with healthy tissues. In particular, the concentration of GSH in the cytoplasm of multidrug-resistant tumors is 4 times greater than in healthy tissues^43^. Furthermore, the cytoplasm of tumor cells contains a higher level of GSH (2–10 mM) than the extracellular matrix (2–20 µM)^44^. Thus, the redox-responsive nanocarriers have attracted increasing attention as a method to selectively deliver therapeutic agents to tumor cells rather than normal cells or healthy tissues because of the high level of GSH in the tumor microenvironment (**Figure 6**). Based on the aforementioned properties of the tumor microenvironment, the redox-sensitive units (diselenide, disulfide, and manganese dioxide) are usually utilized to design redox-responsive nanocarriers^3,45,46^. The biodegradability of redox-responsive nanocarriers has also been achieved in the presence of high intracellular GSH concentrations because of the introduction of redox-sensitive moieties within their networks. Recently, some progress has been achieved in redox-responsive nanocarriers for the effective delivery of the therapeutic agents to tumor cells^32^. Regardless of the approach used to develop these molecules, the redox-responsive nanocarrier has become a focus of attention in the application of drug delivery for cancer treatment.

**Figure 5:**
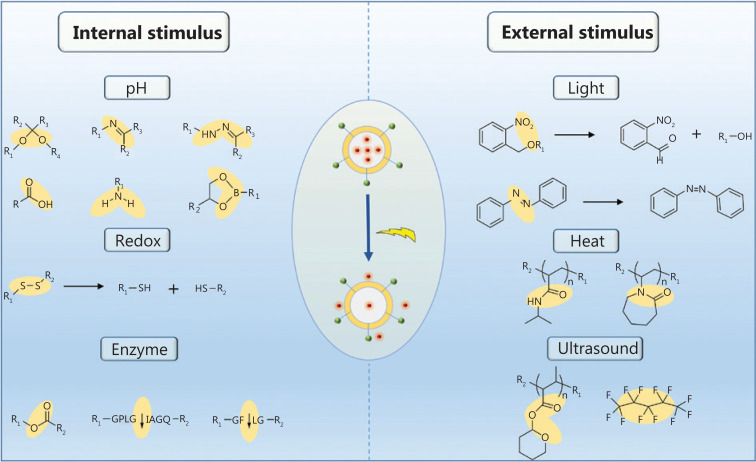
The design of “triggerable” units that respond to internal or external stimuli for the delivery of therapeutic agents.

In redox-responsive nanocarriers, the disulfide bond has attracted increasing attention due to its degradation in the presence of high intracellular GSH concentrations (**Figure 5**). Numerous redox-sensitive micelles containing disulfide bonds have been fabricated in recent years due to their cleavage in response to GSH in the cytoplasm and endolysosomes. The disulfide-cross-linked micelles were prepared using poly (ethylene glycol)*-b-*poly(acrylic acid-*cotert*-butyl acrylate)-poly(ε-caprolactone). This redox-responsive nanocarrier presented excellent biocompatibility, favorable biodegradability, and the ready release of therapeutic agents by responding to the high level of GSH in the tumor microenvironment. Therefore, core-shell-corona nanocarriers are expected to be attractive “smart” redox-sensitive nanosystems for the tumor microenvironment-responsive controlled delivery of therapeutic agents to treat cancer^47^. The diselenide bond is also a dynamic covalent bond that responds to GSH in a sensitive manner^48^. The nanocarriers containing a diselenide bond have attracted increasing attention in drug delivery for cancer therapy in the last few decades. A series of selenium-containing nanoparticles were designed to deliver chemotherapeutic agents. Unlike traditional redox-responsive nanocarriers, these selenium-containing nanocarriers exert potential anticancer effects because of their sensitivity to GSH or other reducing molecules. Meanwhile, these selenium-containing nanocarriers might avoid the premature leakage of chemotherapeutic agents *in vivo* and achieve the selective accumulation of chemotherapeutic agents in tumor tissues^49^.

Manganese dioxide (MnO_2_) has attracted considerable attention in cancer treatment due to its stability in biological fluids and its disintegration by endogenous GSH in the cytoplasm of tumor cells^8,3^. The degradation products generated from MnO_2_ are easily metabolized by human tissues^50^, which contributes to the designation of MnO_2_-based nanocarriers as redox-responsiveness materials. As mentioned above, MnO_2_ is potentially useful as an interlocking agent to endow the polymer-based nanocarrier with a fairly stable structure for drug delivery applications^3^. MnO_2_-based nanocarriers with redox-responsive properties are prepared in 3 steps: (i) complete dissolution of the copolymer segments in an aqueous phase at their initial stage, (ii) a reduction in the solublity of the polyacrylic acid block to drive phase separation during the process of nucleation, and (iii) nucleation of polyacrylic acid/MnO_2_ segments to induce the self-assembly of the copolymer chains at their final stage. After the disintegration of MnO_2_ at tumor sites, this redox-responsive nanocarrier unloads therapeutic agents and is further degraded into biocompatible products compared to other polymer-based nanocarriers. Importantly, this MnO_2_/polymer nanomedicine overcomes the cardiotoxicity of doxorubicin *in vivo*. In our opinion, the redox-responsive nanocarrier represents one of the most efficient methods to increase the anticancer efficacy of nanomedicines by exploiting the tumor microenvironment.

In summary, redox-responsive nanocarriers release chemotherapeutic agents upon exposure to specific redox conditions in their surroundings. In addition to accumulating drugs in the cytoplasm of tumor cells, the redox-responsive nanocarriers also decrease the adverse effects on healthy tissues. Meanwhile, the redox-responsive nanocarriers present favorable biocompatibility and biodegradability during the process of drug delivery. Thus, redox-responsive nanocarriers are the preferred option for drug delivery to treat cancer. Currently, chemotherapy is a topic of extensive research and is widely applied in the clinic. With the rapid development of nanotechnology, the application of redox-responsive nanocarriers in drug delivery will likely receive increasing attention in the future.

## Enzyme-responsive nanocarriers

**Figure 6:**
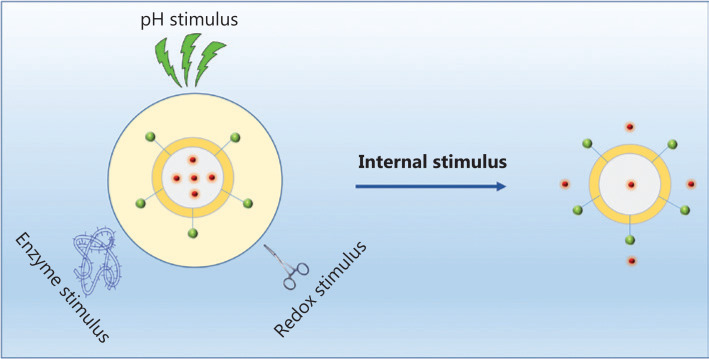
Drug release from stimuli-responsive nanocarriers triggered through internal stimulus.

Compared with normal values of healthy tissues, the concentrations of specific enzymes and proteins, such as prostate-specific antigen, phospholipases, hyaluronidases, matrix metalloproteins, and esterase, are present at much higher levels in tumor tissues because of the abnormalities associated with tumor development^51,52^. Enzyme-sensitive drug delivery systems have attracted increasing attention in recent years due to the enzyme-responsive drug release at the target sites (**Figure 6**)^52,53^. Accordingly, large numbers of enzyme-responsive nanocarriers have been designed, prepared, and implemented for the controlled release of therapeutic agents^52,54^. Currently, the focus on enzyme-responsive nanocarriers involves the release of therapeutic agents at tumor sites. **Figure 5** describes some of the enzyme-sensitive moieties that have been incorporated in enzyme-responsive nanocarriers in recent years. A series of enzyme-responsive nanocarriers exhibited enzyme-triggered cleavage of chemical bonds in the tumor microenvironment, whereas these chemical bonds were relatively stable during circulation in the blood. Enzyme-triggered cleavage of chemical bonds induces the dissociation of enzyme-responsive nanocarriers and subsequent drug release to achieve the goal of cancer treatment.

Short peptides such as enzyme cleavage site-consensus sequences have been utilized within the tumor microenvironment, as shown in **Figure 5**^55,56^. Zhu et al.^57^ reported a typical example of matrix metalloproteinase-sensitive nanocarriers. This external coating was removed by metalloproteinases to improve targeting. In enzyme-responsive nanocarriers, the ester bond has attracted increasing attention due to its degradation in the presence of high esterase concentrations (**Figure 5**). A polymer-based nanocarrier containing boronic esters and *N*-isopropylacrylamide was prepared to load chemotherapeutic agents^58^. Additionally, this polymer-based nanocarrier displayed excellent stability and ensured a long circulation time *in vivo*. Importantly, using this approach, the release of the therapeutic agent was triggered by esterase-mediated cleavage within the tumor microenvironment. Meanwhile, Wu et al.^59^ prepared an enzyme-responsive nanocarrier with a core-shell structure through the self-assembly of the peptide at the surface of silicon nanoparticles. These core-shell nanocarriers achieved the ideal release of chemotherapeutic agents through enzymatic cleavage.

Notably, hyaluronidase (HAase) is another enzyme that is present at high levels within the tumor microenvironment^60^. Thus, hyaluronic acid (HA) is an ideal building block that has recently been applied by various researchers to design HAase-responsive nanocarriers due to their biodegradability, biocompatibility, and active CD44 targeting ability^61,62^. Chen et al.^61^ reported tailor-made hyaluronic acid-based nanocarriers that contained “tetrazole-alkene” photoclick chemistry and microfluidics. These hyaluronic acid-based nanocarriers had strong green fluorescence, high stability, a narrow size distribution, and a defined size. The nanocarriers exhibited the HAase-dependent release of herceptin. Finally, a cumulative release rate of 80.6% of herceptin was observed after an incubation with 1 U/mL HAase for 10 days. Importantly, the released herceptin exhibited antitumor activity, as it maintained its secondary structure in the tumor microenvironment. Using a similar strategy, Bai et al.^62^ also prepared a hyaluronic acid-based nanocarrier containing β-cyclodextrin, hyaluronic acid, and curcumin. This hyaluronic acid-based nanocarrier exhibited esterase-responsive release behaviors, therapeutic efficiency, and an active targeting ability. This enzyme-responsive strategy represents a trend toward the sustained and localized delivery of therapeutic agents from enzyme-responsive nanocarriers for cancer treatment.

## Photo-responsive nanocarriers

**Figure 7:**
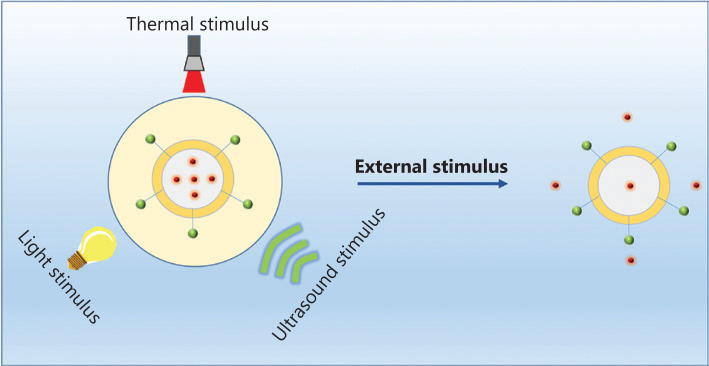
Drug release from stimuli-responsive nanocarriers triggered through external stimulus.

The use of light as an external stimulus promotes the release of therapeutic agents from photoresponsive nanocarriers for cancer treatment (**Figure 7**)^32,63,64^. Importantly, changes in the structures of these photoresponsive nanocarriersoccur upon light irradiation (e.g., photocleavage of the light-responsive units and cis-trans isomerization of azobenzenes)^65,66^. In photoresponsive nanocarriers, photoisomerized and photocleavable units are coupled to the backbone of the polymer. Photocleavage or photoisomerization of light-responsive species leads to the release of the active payload upon irradiation with an external light source [generally ultraviolet (UV) light]. In recent years, most studies of photoresponsive therapy have focused on nanocarriers with a high therapeutic efficiency. The tunable release of therapeutic agents has also stimulated widespread interest^65,66^. **Figure 5** illustrates some examples of photosensitive species developed in recent years, including their structures and phototransformable and photocleavable routes.

UV light is a common stimulus that induces photocleavage and phototransformation of light-responsive species, due to its high energy. In most cases, *o*-nitrobenzyl (*o*NB) is the most commonly used moiety for the photocleavage of the light-responsive unit, and its photocleavage in response to UV irradiation triggers the rapid disassembly of the polymer-based nanocarrier into small molecules. As a drug nanocarrier, this type of photoresponsive nanocarrier shows excellent stability under physiological conditions, but disintegrates rapidly once the photoresponsive units are removed by triggering with UV irradiation. In a representative example, a photosensitive amphiphilic copolymer containing a photocleavable *o*NB unit was prepared through atomic transfer radical polymerization and subsequently self-assembled into polymeric micelles with a photoresponsive property in aqueous solution^65^. By optimizing the number of the photocleavable *o*NB units, polymeric micelles with an average hydrodynamic diameter of 163 nm were obtained as a potential nanocarrier for the UV-triggered delivery of doxorubicin. After UV irradiation for 20 min, a high cumulative DOX release rate of 74.70% from photoresponsive micelles was obtained. The photocleavable *o*NB units were cleaved upon irradiation with UV light, and then the polymeric micelles degraded into water-soluble polymeric products to favor their metabolism.

Photoisomerization represents the most commonly used strategy among the photochemical reaction mechanisms. Photoisomerization induced by UV irradiation has been used to design a photoresponsive nanocarrier for application as a drug delivery system for cancer therapy. Azobenzene (AZO)-based derivatives are extensively used to fabricate photoisomerized nanocarriers^32,63^. The construction of the network of nanocarriers is altered due to the photoisomerization of photoresponsive species upon UV irradiation, which accelerates the release of chemotherapeutic agents and enhances their efficacy against cancer. AZO has attracted increasing attention because of its excellent photoisomerization induced by UV irradiation. The stable *trans* isomer and metastable *cis* isomer are 2 reversible isomers of the AZO under different circumstances. The photoisomerization reaction between the *trans* isomer and *cis* isomer is achieved by exposing the nanocarrier to UV light (λ = 300–400 nm), which is denoted as the *trans-*to-*cis* isomerization of AZO^63^. For example, azobenzene-terminated poly [2-(dimethylamino) ethyl methacrylate] has been used as a photoresponsive unit to design a series of photoresponsive nanocarriers for application in drug delivery^67^. These nanocarriers exhibited good reversibility upon UV irradiation. Upon UV irradiation, the dissociation of the photoresponsive nanocarriers was induced, leading to the increased release of chemotherapeutic agents. As another example, Lu et al.^66^ prepared polymeric micelles based on azide-functional trifluoromethoxy-azobenzene and amphiphilic poly(ethylene glycol)-modified poly(carbonate)s. These polymeric micelles displayed reversible disassembly and self-assembly under UV irradiation due to the properties of AZO. Photoresponsive nanocarriers have been created for drug delivery, and substantial progress has been achieved. Based on the aforementioned studies, the application of photoresponsive nanocarriers is particularly promising for cancer therapy in the near future.

## Thermoresponsive nanocarriers

Thermoresponsive nanocarriers have attracted increasing attention because of their practicality and ease of use in many fields, particularly in drug delivery (**Figure 7**). A low critical solution temperature (LCST) is a key indicator used to estimate the thermoresponsiveness of materials^32^. At a temperature above LCST, the nanocarriers exist in a gel state, while the nanocarriers are in a solution state below the LCST. Hence, the LCST of thermoresponsive nanocarriers should be between body temperature and room temperature, which is suitable for drug delivery. In recent years, some thermoresponsive polymers have been used, including poly(N-vinylcaprolactam) (PVCL) and poly(*N*-isopropylacrylamide) (PNIPAAm)^68,69^.

The most successful thermoresponsive polymer is PNIPAAm, which has been extensively studied since its earliest report in 1967^70^. Upon heating, its hydrophobic-hydrophilic phase transition is observed in aqueous solution because of its response to temperature changes. PNIPAAm exists in a solution state below its LCST, while it is present in a gel state above its LCST. The mechanism of the phase transition is potentially attributed to the hydrogen bonds between amide groups and water. Thus, the phase transition of PNIPAAm has been used to design thermoresponsive nanocarriers for application in drug delivery. As an example, An et al.^71^ prepared a thermoresponsive nanocarrier through the co-assembly of an arm star quaterpolymer and therapeutic agent. The thermoresponsive nanocarrier exhibited the heat-sensitive release of chemotherapeutic agents due to the introduction of PNIPAAm. Importantly, these thermoresponsive nanocarriers also showed increased cellular uptake, and smart release with precise spatiotemporal control obtained with this drug delivery system for cancer therapy.

Poly(*N*-vinylcaprolactam) (PVCL) is another thermoresponsive polymer that has been extensively developed as a drug delivery system for cancer therapy. A continuous coil-to-globule phase transition of PVCL was obtained from 36–50 °C due to differences in the polymer molar mass and concentration, which contributed to controlling the LCST of PVCL through the modulation of the polymer chain length^72^. Thus, the phase transition of PVCL has been used to fabricate thermoresponsive nanocarriers as drug delivery systems for cancer therapy. Kozlovskaya et al.^68^ developed a series of thermoresponsive poly(3-methyl-*N*-vinylcaprolactam)-*block*-poly(*N*-vinylpyrrolidone) diblock copolymers through RAFT polymerization. The LCST ranged from 19.2 °C to 18.6 °C and to 15.2 °C by decreasing the length of hydrophilic segments. Unlike polymeric micelles, these thermoresponsive nanocarriers showed an exceptionally high encapsulation efficiency (95%) and loading capacity for doxorubicin (49%). Importantly, these nanomedicines did not cause death in mice, indicating their potential for use in cancer therapy. The results provide evidence for the safety of these thermoresponsive nanocarriers *in vivo*, suggesting their potential for development into advanced nanomedicines with minimal side effects. These thermoresponsive nanocarriers may show great potential in drug delivery for cancer therapy in the near future.

## Ultrasound-responsive nanocarriers

According to the effects of ultrasound, ultrasound-induced effects on biological tissues are divided into 2 major categories, including nonthermal effects and thermal effects. The nonthermal effects are commonly referred to as cavitation effects. In this case, the ultrasound vibration generates tiny gas bubbles that change the cell membrane permeability and increase the pressure to trigger the release of chemotherapeutic agents from ultrasound-responsive nanocarriers (**Figure 7**)^73^. The thermal effects refer to the conversion of energy, which plays an important role in the transition from acoustic energy to thermal energy. This transition to thermal energy contributes to increasing the temperature of the tissue and the permeability of the vasculature^74^. Furthermore, ultrasound-responsive nanocarriers have an important feature that ensures deep permeation and visualization of tissue. Based on the aforementioned effects, ultrasound-responsive nanocarriers have been extensively developed as drug delivery systems for cancer therapy. Studies exploring ultrasound-responsive nanocarriers for ultrasound therapy have also been a popular research topic in recent years^75^. **Figure 5** illustrates some examples of ultrasound-responsive nanocarriers that have been developed in recent years.

Cavitation effects are widely applied as potent stimuli for improving the drug delivery efficacy in the target tissues. In this respect, a series of therapeutic agents may be incorporated into ultrasound-responsive nanocarriers, and then drug release is triggered by ultrasound. As an example, polymer-based nanocarriers with a narrow distribution were prepared from poly(*N*-(2,2′-dimethylamino) aspartamide, poly(3-acrylamidophenylboronic acid), and poly(ethylene glycol). In this ultrasound-responsive nanocarrier, poly(*N*-(2,2′-dimethylamino) aspartamide was used to effectively encapsulate doxorubicin and perfluoro-n-pentane. Perfluoro-n-pentane is a phase-transitional agent^74^. Because poly(N-(2,2′-dimethylamino) aspartamide dissolves at pH 7.4, the ultrasound-responsive hollow nanospheres containing doxorubicin and phase-transitional perfluoro-n-pentane were loaded into the aqueous lumen^76^. The ultrasound-responsive nanocarriers were destroyed by the external application of low-frequency ultrasound due to the liquid-to-gas phase transition of perfluoro-n-pentane, and then rapid drug release from ultrasound-responsive nanocarriers occurred to achieve drug delivery deep inside a tumor^76^.

On the other hand, the energy generated by acoustic vibrations not only causes an increase in the local temperature but also controls drug release from ultrasound-responsive nanocarriers (**Figure 7**). In one representative example, an ultrasound-sensitive NB bearing siRNA (siRNA-NB) for tumor therapy was prepared *via* a heteroassembling strategy using the siRNA-complexed polymeric micelles and gas-cored liposomes to serve as an ultrasound-responsive nanocarrier for cancer therapy^77^. The ultrasound-responsive nanocarrier effectively enhanced the gene silencing effect of siRNA-NBs, which resulted in a substantial increase in cancer cell apoptosis. Furthermore, a significantly improved therapeutic effect was obtained *in vivo*^77^.This ultrasound-responsive nanocarrier may be a desired vehicle to deliver the active payload for cancer therapy. In addition to the direct delivery of therapeutic agents, several investigators have reported a crucial role of the sonosensitizer in sonodynamic therapy^42,78^. As an example, porous silicon nanoparticles have been reported to serve as potential sensitizers for ultrasound-assisted therapy, which exhibited a strong suppression of cancer cell proliferation upon exposure to ultrasound^42^.

Although substantial progress has been achieved in ultrasound-responsive nanocarriers for drug delivery, some challenges remain to be addressed to improve the therapeutic efficacy. First, the thermal effects generated by acoustic vibrations should be further explored to avoid localized overheating, resulting in heat-induced damage to biological tissues. However, ultrasound-responsive nanocarriers have primarily been analyzed *in vivo*, including the biocompatibility, biodegradability, and pharmacokinetics of ultrasound-responsive nanocarriers and tissue permeation studies. Despite these challenges, ultrasound-responsive nanocarriers will likely attract increasing attention in the near future due to their unique cavitation effects and thermal effects.

## Nanomedicines in clinical cancer care

Several nanomedicines have been used in clinics and clinical trials, including polymer and hybrid-based nanocarriers. Various types of nanomedicines have been investigated in phase 1 trials in patients or in advanced (phases 2 and 3) clinical trials. Some nanomedicines under investigation in clinical studies or approved for clinical cancer care are summarized in **Table 1**.

**Table 1:** Examples of nanocarriers in clinics and clinical trials

Product	Drug	Nanocarrier	Application
In clinics			
ADI-PEG 20	Arginine deaminase	Polymeric	Hepatocellular carcinoma
Doxil	Doxorubicin	Polymeric	Leukaemia,lymphoma, and carcinoma
AP5280	Platinum	Polymeric	Solid tumors
DepoCyt	Cytarabine	Liposomal	Lymphomatous meningitis
MAG-CPT	Camptothecin	Polymeric	Solid tumors
Visudyne	Verteporfin	Liposomal	Macular degeneration
Oncaspar	L-Asparaginase	Polymeric	Lymphoblastic leukemia
Pegasys	Interferon alfa-2a	Polymeric	Hepatitis B and hepatitis C
Clinical trials			
PNU166945	Paclitaxel	Polymeric	Solid tumors
Lipoplatin	Cisplatin	Liposomal	Non-small cell lung cancer
XMT-1001	Camptothecin	Polymeric	Gastric cancer and lung cancer
Onco-TCS	Vincristine	Liposomal	Relapsed non-Hodgkin lymphoma
OSI-211	Lurotecan	Liposomal	Head, neck and ovarian cancer
SPI-077	Cisplatin	Liposomal	Head, lung and neck cancer
PEG-SN38	Irinotecan derivate	Polymeric	Solid tumors and breast cancer
Livatag	Doxorubicin	Polymeric	Liver cancer
NKTR-105	Docetaxel	Polymeric	Solid tumors and ovarian cancer
Paclical	Paclitaxel	Polymeric	Breast, lung and ovarian cancer
PEG-docetaxel	Docetaxel	Polymeric	Solid tumors

Polymer-based nanocarriers are another interesting group of drug delivery systems that change the pharmacokinetic profile of a drug. **Table 1** summarizes polymer-based nanocarriers that have been investigated in clinical trials. For instance, the PEG-PGA polymeric micelle NC-6004 (Nanoplatin^®^) containing cisplatin was investigated in phase 1 trials^79^. NC-6004 induced less neurotoxicity than free cisplatin. A phase 3 clinical trial in patients is underway. As another example, NK-105, a polymer-based nanocarrier, is being tested in clinical trials^80^.

Several other polymer-based nanocarriers have been investigated in phase 1 trials, including mitoxantrone-loaded polymeric nanoparticles^81^ and epirubicin-loaded polymeric micelles^82^. Although there have been substantial advances in polymer-based nanocarriers in clinical trials, no hybrid-based nanocarriers have been approved. As an important example, pegylated colloidal gold-TNFα particles are undergoing early clinical testing.

## Toxicity concerns

Advances in polymer-based nanocarriers require safety issues for human health to be addressed. A number of investigators have documented potential detrimental interactions of nanocarriers in cancer therapy. These results have led to the emergence of nanotoxicology as an independent field of research^83^. Considerable effort has been devoted to developing methods for optimizing the polymer to reduce toxicity. Nonetheless, a comprehensive comparison of the safety of polymer-based nanocarriers with other nanocarriers has not been conducted. An assessment of the acute toxicity of polymer-based nanocarriers is more demanding, and data are largely missing^84^. With the development of nanotechnology, the toxicity of polymer-based nanocarriers may be minimized by combining more predictive diagnostic tools with novel targeting strategies. Individualized cancer therapy based on the polymer-based nanocarriers can be achieved, which will show huge potential in the future.

## Summary and challenges

Polymer or hybrid-based nanocarriers have become a research hotspot in drug delivery due to the development of nanotechnology and the emergence of new functional materials in recent years, particularly for cancer treatment. Numerous researchers have focused on fabricating functional nanocarriers for the delivery of therapeutic agents^85–87^. Although substantial advances in hybrid nanocarriers have been reported, polymer-based nanocarriers are being extensively investigated and have achieved much success in clinics. Specifically, an efficient and successful nanocarrier must: 1) avoid the premature leakage of therapeutic agents during circulation in the blood under physiological conditions, 2) possess targeting ability, to accumulate at the tumor sites to provide a sufficient dose of therapeutic agents and reduce the severe side effects of therapeutic agents on healthy tissues, show biocompatibility, and 3) exhibit biodegradability.

Nanocarriers, particularly polymer or hybrid-based nanocarriers, have received increasing attention due to their biodegradability, biocompatibility, structural stability in physiological medium, and structural instability at tumor sites. A tremendous amount of effort has been devoted to the development of efficient polymer or hybrid-based nanocarriers with stimuli-responsive properties. This review has summarized recent advances in pH-, redox-, enzyme-, photo-, thermo-, and ultrasound-responsive nanocarriers that achieved drug delivery to the tumor. In terms of cancer treatment, nanocarriers not only effectively encapsulate and deliver chemotherapeutic agents, but also induce localized release at tumor sites. Notably, the hybrid-based nanocarriers have also been used in imaging to achieve greater therapeutic efficacy. A combination of diagnosis and treatment will likely be promoted based on the rapid development of hybrid-based nanocarriers, which will lead to the enhancement of theranostics in the field of tumor treatment. As mentioned above, hybrid-based nanocarriers incorporating theranostic features have been designed and fabricated to confirm their great potential for achieving the accurate treatment of cancer in the future.

Nanomedicine is one of the most promising strategies being developed in the frontier of cancer therapy. The widespread use of nanomedicines requires a comparative and quantitative analysis of their therapeutic effects. An increasing amount of data is becoming available on the therapeutic effect of nanomedicines used in cancer therapy. Nonetheless, a comparison of the therapeutic effects of different nanomedicines remains a challenge. Thus, the assessment of the therapeutic effect of nanomedicines may be inadequate at present, and the development of assays comparing the therapeutic effects of different nanomedicines should be encouraged. The lack of data renders the comparative and quantitative analysis of different nanomedicines and their consequences more challenging, but also more important.

Nonetheless, dilemmas or challenges in the design of efficient polymer or hybrid-based nanocarriers with stimuli-responsiveness properties still exist. First, regardless of the progress that has been reported, both the structural stability of nanocarriers in a physiological medium and the structural instability of nanocarriers at tumor sites is difficult to achieve. Although substantial advances in hybrid-based nanocarriers have been documented, the discovery of new nanocarriers still hinders the development of nanomedicines. In addition, in the last few decades nanocarriers have been commonly overdesigned to combine many functions in one molecule to generate a multifunctional nanomedicine. These overdesigned nanocarriers are often too complex and prevent translation from the experimental stage to clinic applications. Nature always operates according to the simplest and most economical principles. As a result, simple polymer or hybrid-based nanocarriers with a cost-effective synthesis route that are easy to scale up will likely be the most useful. Ultimately, these dilemmas and challenges will spur biomedical scientists to understand the interactions between the structure and function of nanocarriers and improve the design of nanocarriers. Therefore, joint efforts by scientists from multiple disciplines will subsequently expedite the realization of the potential of hybrid-based nanocarriers with stimuli-responsive properties for cancer therapy.
